# Effects of Cage Enrichment on Behavior, Welfare and Outcome Variability in Female Mice

**DOI:** 10.3389/fnbeh.2018.00232

**Published:** 2018-10-26

**Authors:** Jeremy D. Bailoo, Eimear Murphy, Maria Boada-Saña, Justin A. Varholick, Sara Hintze, Caroline Baussière, Kerstin C. Hahn, Christine Göpfert, Rupert Palme, Bernhard Voelkl, Hanno Würbel

**Affiliations:** ^1^Division of Animal Welfare, Veterinary Public Health Institute, University of Bern, Bern, Switzerland; ^2^Division of Livestock Sciences, Department of Sustainable Agricultural Systems, University of Natural Resources and Life Sciences Vienna (BOKU), Vienna, Austria; ^3^Institute for Animal Pathology, University of Bern, Bern, Switzerland; ^4^Department of Biomedical Sciences, University of Veterinary Medicine Vienna, Vienna, Austria

**Keywords:** environmental enrichment, variation, animal welfare, mice, behavioral phenotypes

## Abstract

The manner in which laboratory rodents are housed is driven by economics (minimal use of space and resources), ergonomics (ease of handling and visibility of animals), hygiene, and standardization (reduction of variation). This has resulted in housing conditions that lack sensory and motor stimulation and restrict the expression of species-typical behavior. In mice, such housing conditions have been associated with indicators of impaired welfare, including abnormal repetitive behavior (stereotypies, compulsive behavior), enhanced anxiety and stress reactivity, and thermal stress. However, due to concerns that more complex environmental conditions might increase variation in experimental results, there has been considerable resistance to the implementation of environmental enrichment beyond the provision of nesting material. Here, using 96 C57BL/6 and SWISS female mice, respectively, we systematically varied environmental enrichment across four levels spanning the range of common enrichment strategies: (1) bedding alone; (2) bedding + nesting material; (3) deeper bedding + nesting material + shelter + increased vertical space; and (4) semi-naturalistic conditions, including weekly changes of enrichment items. We studied how these different forms of environmental enrichment affected measures of animal welfare, including home-cage behavior (time–budget and stereotypic behavior), anxiety (open field behavior, elevated plus-maze behavior), growth (food and water intake, body mass), stress physiology (glucocorticoid metabolites in fecal boluses and adrenal mass), brain function (recurrent perseveration in a two-choice guessing task) and emotional valence (judgment bias). Our results highlight the difficulty in making general recommendations across common strains of mice and for selecting enrichment strategies within specific strains. Overall, the greatest benefit was observed in animals housed with the greatest degree of enrichment. Thus, in the super-enriched housing condition, stereotypic behavior, behavioral measures of anxiety, growth and stress physiology varied in a manner consistent with improved animal welfare compared to the other housing conditions with less enrichment. Similar to other studies, we found no evidence, in the measures assessed here, that environmental enrichment increased variation in experimental results.

## Introduction

The mouse, *Mus musculus*, is the most widely used animal in research, and yet surprisingly little is known about how its behavioral biology relates to the social and physical aspects of current laboratory housing conditions (Latham and Mason, 2004). Consequently, the Committee updating the US Guide for the Care and Use of Laboratory Animals identified a critical lack of empirical evidence on “space and housing needs” and “enrichment, exercise and human contact” (Garber, 2011; National Research Council, 2011). We have recently reported on a systematic assessment of the effects of space allowance on measures of animal welfare in laboratory mice (Bailoo et al., 2018); here we report on the effects of cage enrichment on behavior and measures of welfare in female laboratory mice.

Housing conditions for laboratory mice have been shaped primarily by economics (minimal use of space, equipment and labor), ergonomics (ease of handling, visibility of animals), hygiene (easy to sanitize) and standardization (minimization of variation; see Olsson and Dahlborn, 2002; Baumans and Van Loo, 2013, for further details). Typically, mice have been kept in transparent “shoe-box” cages with bedding, food and water. Such housing conditions lack sensory and motor stimulation and may prevent mice from performing species–typical behaviors, such as nest building (Würbel, 2001; Latham and Mason, 2004). Consequently, such housing conditions are associated with signs of impaired welfare, including abnormal repetitive behavior (Garner et al., 2004a,b, 2011; Garner, 2005; Würbel, 2006; Gross et al., 2012) and anxiety (Chapillon et al., 1999; van Praag et al., 2000; Würbel, 2001). Based on such evidence, Switzerland has declared environmental enrichment (i.e., nesting material) mandatory (The Swiss Federal Council, 2008), while the EU Directive 2010/63/EU (European Parliament Council, 2010) and the US Guide for the Care and Use of Laboratory Animals (2011) only recommend enrichment of rodent cages. Thus, an increasing number of researchers are using nesting material, shelters, gnawing sticks and other enrichment items with the goal of improving the welfare of laboratory rodents (The Swiss Federal Council, 2008; European Parliament Council, 2010; Baumans and Van Loo, 2013).

Environmental enrichment is used to increase sensory and motor stimulation, to facilitate species–typical behavior, and to provide the animals with some degree of control over their environment (Dawkins, 1988; Newberry, 1995; Olsson and Dahlborn, 2002; Nithianantharajah and Hannan, 2006; Gross et al., 2011b; Bennett et al., 2018). Current evidence indicates that even the addition of simple forms of enrichment to standard laboratory cages can improve the welfare of laboratory mice, as shown by reduced abnormal repetitive behavior (DeLuca, 1997; Würbel et al., 1998; Nevison et al., 1999a; Latham and Mason, 2010; Tilly et al., 2010; Bechard et al., 2011; Gross et al., 2011b, 2012) and reduced measures of anxiety (e.g., Chapillon et al., 1999; Roy et al., 2001; Benaroya-Milshtein et al., 2004; Binder et al., 2004; Görtz et al., 2008; Sztainberg and Chen, 2010), although not all studies have confirmed these findings (van de Weerd et al., 1994; Nevison et al., 1999a; Zhu et al., 2006).

In most studies, cage enrichment adds several resources, environmental complexity and sometimes novelty to the standard housing conditions. However, one study has found that within standard laboratory cages neither complexity nor novelty of enrichments had beneficial effects beyond those of nesting material alone (Gross et al., 2011b). Arguably, nesting material is the only enrichment that has consistently been found to be beneficial for mouse welfare (for review, see Olsson and Dahlborn, 2002).

Nesting material is an important resource for laboratory mice as it allows for the expression of species–typical nest building behavior, facilitates thermoregulation and provides shelter, if provided in sufficient quantity (Bult and Lynch, 1997; Sherwin, 1997; van de Weerd et al., 1997; Olsson and Dahlborn, 2002; Smith and Corrow, 2005; Gaskill et al., 2009, 2011, 2012; Gross et al., 2011b). Mice are highly motivated to construct nests (Nicol et al., 2008), and prefer nesting material to nest boxes (van de Weerd et al., 1998). Furthermore, for resting and maintenance behavior, mice prefer much higher ambient temperatures (30–32°C) than are common in animal facilities (20–26°C)—without nesting material, laboratory mice are at a higher risk for experiencing chronic cold stress (e.g., Johnson et al., 2017).

Other forms of enrichment, by contrast, have produced inconsistent effects (for reviews, see Jennings et al., 1998; Olsson and Dahlborn, 2002; Benefiel et al., 2005; Smith and Corrow, 2005; Whittaker et al., 2012; Baumans and Van Loo, 2013). For example, in some studies the provision of shelters has been associated with increased levels of aggression in male mice (McGregor and Ayling, 1990; Haemisch et al., 1994; Howerton et al., 2008), but this seems to depend strongly on the strain of mouse (van de Weerd et al., 1994; Chapillon et al., 1999; Nevison et al., 1999a).

The aim of the present study was to replicate and extend the results of previous studies on the effects of cage enrichment on mouse behavior and measures of welfare. We varied cage enrichment across four levels, including cages: (i) without any enrichment (Barren, B); (ii) with nesting material (Nesting, N); (iii) with deep bedding, shelters and additional vertical space besides nesting material (Enriched, E); and (iv) large pet cages attached to a laboratory cage, offering multiple resources and different items each week for active engagement (Super-Enriched, SE). To increase the generality of our findings, we studied an inbred and an outbred strain of mouse. However, similar to other proof-of-concept studies on environmental enrichment in mice (Van Loo et al., 2003; Wolfer et al., 2004), we only studied female mice as some forms of enrichment are associated with escalating aggression in male mice. Our primary outcome measures of animal welfare were stereotypy performance in the home–cage and measures of anxiety in behavioral tests, as these two measures were most consistently found to be improved by environmental enrichment. Additionally, a range of secondary outcome measures covering different domains of animal welfare were also included: home-cage behavior, measures of growth, endocrine stress responses, brain function, and emotional state. We tested the hypothesis that the welfare of mice increases with increasing degrees of cage enrichment across our four treatment groups, following the prediction: B < N < E < SE.

Since environmental enrichment renders the animals’ environment more complex, concerns have been raised that cage enrichment might lead to higher variability in experimental results. Although empirical evidence does not support these concerns (Augustsson et al., 2003; Wolfer et al., 2004; Baumans et al., 2010; van de Weerd et al., 2010) they seem to persist (e.g., Toth et al., 2011). Therefore, we additionally assessed how cage enrichment affected variability in all measured outcomes.

## Materials and Methods

### Experimental Design, Animals and Housing Conditions

We used a 4 (cage enrichment) × 2 (mouse strain) factorial design conducted in two consecutive batches of equal size; the second batch began 3 weeks after the end of the first batch. Subjects were 192 female mice, 96 each of the inbred strain C57BL/6JRj (C57) and the outbred strain RjOrl:SWISS (SWISS) from Janvier Labs, France. Each batch was comprised of 48 newly weaned non–sibling mice (21–25 days old at delivery) per strain. All mice were ear-tattooed for identification by the same two experimenters (JB and EM) on the day following arrival at the laboratory.

A review of the literature, comparing variation in behavioral outcomes between barren cages and cages with nesting material alone, yielded an effect size range of 0.85–1.0 (Cohen’s *d*) resulting in a sample size of *n* = 12 per treatment group. Expecting some of our behavioral measures, such as the behavior in the elevated plus–maze, to be more variable and thus yield a smaller effect size, and to accommodate for possible attrition, we adjusted our sample size to *n* = 24 per experimental condition per strain.

Mice were randomly allocated to the four housing conditions, B, N, E and SE, described in further detail in Supplementary Table S1. They were housed in groups of three per cage, with four replicate cages per strain and treatment in each batch (see Supplementary Table S2). Each batch of mice was delivered in four boxes, two per strain, containing 24 animals each. Each box of mice was allocated to cages and treatment groups sequentially to minimize individual differences in behavior between cages. Animals were housed in two housing rooms, located on either side of the test room (Supplementary Figure S1), with half of the animals per strain per batch in each room. Cage height on the rack was counterbalanced by strain and room between batches.

The B, N and E housing conditions consisted of a Makrolon^®^ Type three cage, which besides food (Kliba Nafag #3430, Switzerland) and tap water *ad libitum*, contained either bedding only (B; Lignocel^®^ select, see Supplementary Table S1 for depth), bedding and nesting material (N), or deeper bedding, nesting material, a tunnel, a shelter and increased vertical space (E). To increase the generality of our findings, we used two different types of nesting material (10 g of Sizzle Pet^®^ or three paper tissues), tunnels (rat tunnel Plexx EU #13104 or rat retreat Plexx EU #13154) and shelters (arch Plexx EU #13244 or hut Plexx EU #13169), counterbalanced across cages.

The SE housing condition consisted of a Makrolon^®^ Type three cage connected to a Savic™ Mickey XL pet cage, by a clear polycarbonate tunnel 6 cm in diameter. The layout of the SE system and the enrichments used are displayed in Supplementary Figures S2, S3. Briefly, the Type three cage contained a paper tunnel (Plexx EU #14152), a paper shelter (Plexx EU #13244), and nesting material (10 g Sizzle Pet^®^ and 3× paper tissues). The Mickey XL cage contained an elevated platform made of polycarbonate (410 cm^2^ in floor area, with a 5 cm wall), with a wooden ladder leading up to it and a wooden shelter on top of it. A plastic hammock was attached to the cage lid above the elevated platform where a secondary source of food and water was also accessible. Two wooden coconuts attached to the cage lid and joined by a suspended bridge made of rope and wood, a paper shelter, and a paper tunnel were also provided. These items in the Mickey XL cage were present throughout the study. To stimulate activity and exploration further, and to increase behavioral diversity, additional items were rotated in the SE cage on a weekly basis (see Supplementary Table S3).

### Husbandry Procedures

Animals were kept on a reversed 12:12 light/dark cycle with lights on at 19:00 h. A red light emitting diode (LED) remained on throughout the entire cycle. Temperature was maintained at 22 ± 1°C, with an average humidity of 40%. Husbandry procedures were conducted weekly (see Supplementary Methods).

Because some outcome measures involved long periods of testing and manipulation by the experimenter, all mice were habituated to being handled beginning upon arrival at the laboratory (see Supplementary Methods).

### Outcome Variables

**Figure 1:**
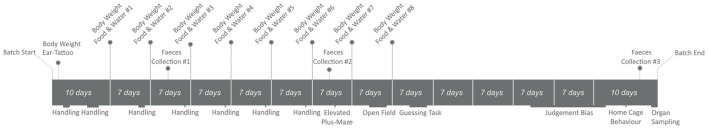
Experimental timeline for a single batch of animals.

Outcome variables covered a range of measures related to animal welfare. Our primary outcome measures were: (i) stereotypy performance in the home–cage; and (ii) measures of anxiety in two behavioral tests (elevated plus–maze test and open field test). In addition, we assessed a range of secondary outcome measures covering different domains of animal welfare, including measures of: (i) home–cage behavior (time budget, use of enrichments); (ii) growth (food and water intake, body weight); (iii) endocrine stress responses (glucocorticoid metabolites in fecal boluses, adrenal weight); (iv) brain function (inhibitory control of behavior as measured by recurrent perseveration in a two–choice guessing task); and (v) emotional state (judgment bias in a spatial Go/No-Go task; Figure 1).

For assessing recurrent perseveration and judgment bias, one focal animal from three of the four cages per treatment, strain and batch were randomly selected (*n* = 48 in total). Cage, treatment and strain were counterbalanced across three experimenters (EM, MB-S, JB) such that: (1) no two experimenters tested animals from the same treatment at the same time; (2) no experimenter tested animals from the same treatment consecutively; and (3) no experimenter tested animals from the same strain consecutively. For judgment bias testing, one experimenter was replaced (MB-S by SH) towards the end of the training period due to an emergency leave of absence in batch 1, and for batch 2, mice from one experimenter (EM) were shaped for two sessions by the other two experimenters (JB and MB-S) due to illness.

#### Stereotypy Performance in the Home–Cage and Other Home–Cage Behavior

Cage enrichment has previously been reported to reduce abnormal repetitive behavior (DeLuca, 1997; Würbel et al., 1998; Nevison et al., 1999b; Tilly et al., 2010; Bechard et al., 2011), and one study has found that nesting material is associated with a reduction of stereotypic behavior in particular (Gross et al., 2012). To evaluate the effects of increasing degrees of enrichment on the incidence of stereotypic behavior, all cages were recorded for 24 h each, prior to the end of the experiment.

Video-recordings were scored separately for stereotypic behavior and other home–cage behavior using Noldus Observer XT (version 10.5) by EM, CB and JB. From each cage all animals were observed and their behavior scored, although, to evaluate the relation between stereotypic behavior and recurrent perseveration, data from focal animals were used (the same ones tested in the guessing task). Videos of two E cages (all intervals) and one SE cage (one interval) were unavailable for scoring due to equipment failure (see below for a description of the intervals).

Stereotypic behavior was scored using a previously validated ethogram (Novak et al., 2016; see also Supplementary Table S4). Mice were observed for 15 min, within four 1-h time windows, distributed across the dark phase (07:30–08:30, 09:30–10:30, 12:30–13:30 and 15:30–16:30). These time windows had been determined by pilot observations to represent two time intervals of high activity and two of low activity. Stereotypic behavior and general activity were sampled using one–zero sampling with 15 s intervals, yielding 240 data points per mouse. Stereotypy performance is reported here as a proportion of active time. Ten percent of all videos were rescored for assessment of intra- and inter-rater reliabilities, which were high throughout (Jansen et al., 2003), *κ* = 0.94 and 0.96, respectively.

In addition to stereotypic behavior, we also assessed the extent to which the mice engaged with the cage environment, including engagement with enrichments, and how the provided resources within the cage affected general daily activities and facilitated species–typical behavior (Supplementary Table S5; category, environmental manipulation). Of note, in terms of engagement with the cage environment, we evaluated manipulation of bedding (e.g., digging) in the B cage, while we additionally evaluated engagement with the provided enrichment items in the N, E and SE cages. Thus, engagement with the cage environment was quantified even in the barren cage, where no enrichments were present. We did not categorize time spent on the lid as engagement with the environment, although these data were analyzed and are described in “Anxiety in the Elevated Plus–Maze Test and Open Field Test” section in our analysis of time budget.

To evaluate engagement with the cage environment, we used the ethogram described under Supplementary Table S5. Mice were observed for 30 min, within six 1-h time windows, four in the dark phase (the same as above) and two in the light phase (20:30–21:30, 03:00–04:00), using instantaneous sampling at 1-min intervals and yielding 180 data points per mouse. Ten percent of all videos were rescored for assessment of intra- and inter-rater reliabilities, which were high throughout (Jansen et al., 2003), *κ* = 0.85 and 0.90, respectively.

#### Anxiety in the Elevated Plus–Maze Test and Open Field Test

Besides reduced stereotypy performance, cage enrichment in mice has most consistently been associated with reduced measures of anxiety in behavioral tests such as the elevated plus–maze test or the open field test. Both elevated plus–maze behavior (Pellow et al., 1985; Carola et al., 2002) and open field behavior (Denenberg, 1969; Carola et al., 2002) have been validated for differences in anxiety in both rats and mice. On the elevated plus–maze, less time spent on, and fewer entries into, the open arms, as well as reduced locomotion reflect higher levels of anxiety (Walf and Frye, 2007). In the open field, longitudinal assessment of the pattern of locomotor behavior in the open field across repeated exposures has been demonstrated to provide information about how animals behaviorally cope with a stressor and how the hypothalamus–pituitary–adrenal (HPA) axis differentially operates between groups of animals (Whimbey and Denenberg, 1967; Bailoo et al., 2013). Specifically, reduced levels of habituation (i.e., reduced exploration and/or time in the center across days of testing) and/or increased levels of sensitization (i.e., greater exploration and/or time in the center across days of testing), between groups of animals, are associated with increased anxiety and vice versa.

In the present experiment, we used three elevated plus–mazes and three open field arenas. Elevated plus–mazes were made of polycarbonate with infrared (850 nm) backlit floors. Each maze consisted of four arms, each 30 cm in length and 6 cm wide, and a center square measuring 6 × 6 cm. Two arms opposite to each other were open, with a small lip around the perimeter 0.5 cm high, while the remaining two arms were enclosed, with walls 15 cm high. The open field arenas were made of polycarbonate with dimensions 45 × 45 × 45 cm^3^ with infrared (850 nm) backlit floors.

For both tests, mice from each cage were randomly assigned to one of three experimenters (EM, MB-S and JB). The test order of cages was counterbalanced by treatment and strain in blocks of four across eight blocks, whereby all four treatments were represented in each block, with two treatments per strain. For consecutive blocks, the strain associated with treatment in the previous block was alternated.

Behavior on the elevated plus–maze was assessed in a single session while behavior in the open field was assessed across four consecutive days; all tests were conducted between 10:00 h and 13:00 h. The test duration for both tests was 5 min. The outcome variables of interest in the elevated plus–maze test were: (1) distance traveled; and (2) time spent in the open arms. In the open field test changes in: (1) distance traveled; (2) time in the center; and (3) time in the corners, across 4 days of testing was evaluated.

For each test session, the cage to be tested was brought to the test room and the overhead lights (120 lux) were turned on. The three animals were then removed from their cage and placed into the apparatus by the assigned experimenter. At the end of the test, the animals were replaced into the home–cage, and the cage returned to the housing room. Between test sessions, the arenas were cleaned with 70% isopropanol. Outcome measures were scored live by Noldus EthoVision XT (version 11.5). Accuracy of video tracking was subsequently evaluated by JB from video recordings ensuring issues associated with automated tracking were eliminated (Bailoo et al., 2010). The detection settings for tracking were selected so that both the percentage of samples in which the subject was not found and the percentage of samples skipped were less than 1% per trial.

#### Growth: Body Weight and Food and Water Intake

Longitudinal assessment of food and water intake and body weight can provide information about the different types of experienced stress, since acute stress is associated with a loss of and chronic stress with a gain in body weight (Klok et al., 2007; McEwen, 2007; Torres and Nowson, 2007; Lutter et al., 2008). Food and water intake are coupled and thus expected to be positively correlated.

Body weight of each mouse and food and water intake at the cage level, were recorded at weekly cage changes. Food and water intake were assessed for each cage by subtracting the weight of the remaining food and water from that of food and water provided at the last cage change. Thus, we measured food disappearance rather than food intake, as particulates of food dropped in the bedding and spilled water remained unaccounted for. For analysis, food and water intake were corrected for the number of animals per cage when there were fewer than three animals in the cage (see “Attrition” section, for further details).

#### Endocrine Stress Responses: Fecal Glucocorticoid Metabolites and Adrenal Weight

Non-invasive methods of quantifying circulating levels of glucocorticoids, a primary product of the activation of the HPA stress system, are preferable to invasive methods such as blood sampling, because they do not elicit a stress response. In mammals, glucocorticoids are metabolized by the liver and are excreted in both urine and feces. A validated method of analyzing glucocorticoid metabolites in mice (Touma and Palme, 2005) was used as a measure of endocrine stress responses prompted by the different housing conditions.

Feces were collected, with a minimum of six boluses per mouse per cage, at three time points in the dark phase under red light approximately 24 h after cage changes. Because the transit time of by-products of corticosterone secretion in feces is between 8 h and 10 h (Touma et al., 2003; Touma and Palme, 2005), it is important to note here that we are measuring, in part, arousal/stress as a consequence of the husbandry procedures associated with cage change (Balcombe et al., 2004). Samples were immediately frozen at −20°C and later processed (blinded to experimental treatment, JV and RP) to assess the concentration of 5α–3β, 11β–corticosterone metabolites (ng/0.05 g feces) as described by Touma et al. (2003, 2004). In total 563 samples were processed; four samples were missing due to attrition (see “Attrition” section), and nine samples were not processed because of insufficient sample material.

At the end of the experiment, animals were killed by anesthesia with 5% isoflurane followed by asphyxiation with CO_2_, performed by JB. Within 2 min, the animals were transported to the dissection laboratory. Dissections were performed by KH and CG. Animals were first weighed, then the adrenals were extracted and weighed using a precision scale to the nearest 10,000th of a gram (Mettler AE 160). Chronic exposure to stress has been associated with higher levels of circulating glucocorticoids and heavier adrenal glands (van de Weerd et al., 1994; McEwen, 2007). Other organs (brain, heart, kidney, liver, spleen) were also extracted and weighed using a precision scale to the nearest thousandth of a gram (Mettler Toledo ME 802) to obtain additional outcome measures to assess treatment-dependent variability in the data. Organ weights were corrected by body weight.

#### Brain Function: Recurrent Perseveration in a Two–Choice Guessing Task

The expression of stereotypic behavior has been found to correlate with recurrent perseveration, a form of impaired inhibitory control of behavior, both in humans (autistic children) and in captive mammals and birds (Turner, 1997; Garner et al., 2003, 2011; Gross et al., 2012). A positive correlation between stereotypy levels and recurrent perseveration has also been observed in mice, notably in C57 mice (Garner et al., 2011), but other studies have yielded mixed results (Latham and Mason, 2010; Gross et al., 2011a, 2012; Novak et al., 2016).

To assess recurrent perseveration, we used a slightly modified apparatus and procedure to the one described previously (Novak et al., 2016; Bailoo et al., 2018). Briefly, all focal mice (*n* = 48) were trained and tested under red light in three virtually identical apparatuses. Each apparatus consisted of a rectangular arena with two goal-holes at one end and a trapezoid-shaped start-box at the opposite end (see Supplementary Figure S4). Animals were first habituated to the apparatus across 3 days and then shaped to retrieve rewards (BioServ chocolate pellets, 20 mg) from both goal holes. Animals were then tested across 2 days, 60 trials/day, where one of the two goals was rewarded with a probability equaling the proportion of responses to the other side in the previous 20 trials (see Novak et al., 2016; see Bailoo et al., 2018 and Supplementary Figure S5 for further details). Perseveration score (logit [P]), as well as the frequencies of pure repetitions (LLLL, RRRR) and pure alternations (RLRL, LRLR) relative to all possible tetragrams of consecutive choices (*n* = 16) were calculated for each individual and analyzed.

#### Emotional State: Judgment Bias in a Spatial Go/No–Go Task

One approach to evaluate the valence (i.e., the positivity or negativity) of emotions in animals is to investigate how decisions in ambiguous situations are biased by the underlying emotional states of the animals—as assessed by cognitive judgment bias tasks (Harding et al., 2004). Previous research evaluating judgment biases in rats indicates that the transfer from standard housing conditions to enriched environments is associated with a relative shift from “pessimistic” to “optimistic” judgments based on the expectancy of non–reward and reward, respectively (e.g., Brydges et al., 2011; Richter et al., 2012). However, no study has investigated the effect of environmental enrichment on judgment biases of mice.

To assess judgment biases, we developed a task which integrates active trial initiation into a spatial Go/No Go task (see Hintze et al., 2018 for further details). Briefly, all focal mice (*n* = 48), were trained and tested under red light in three virtually identical apparatuses (see Supplementary Figure S6). Each apparatus consisted of a rectangular arena with a row of nine goal–holes at one end (five of which were used for this task), and a trapezoid–shaped area containing a nose-poke at the opposite end. Mice were first trained to initiate each trial by nose-poking, and in several subsequent steps, were trained to discriminate that the location of an open goal-hole (at the extreme ends) signaled either reward (Go trial) or non-reward (No–go trial). Once animals had learned this discrimination, ambiguous test trials with open goal–holes at three equidistant intermediate locations between the extreme ends, were interspersed among Go and No–Go trials, across six test sessions. The Go: No–go response ratio to all five goal–holes (positive, three ambiguous, negative) was used as a measure of judgment bias.

### Attrition

In batch 1, one C57 mouse housed in the B condition was euthanized prior to the end of the experiment because the animal was favoring the right side of its head (lopsided tilt). Behavioral symptoms indicated mild distress, although post-mortem pathology yielded no diagnosis. In batch 2, one C57 mouse allocated to E condition was found dead at delivery.

### Ethical Statement

This study was carried out in accordance with the guidelines of the Swiss Animal Welfare Ordinance (TSchV 455.1). It was approved by the Cantonal Veterinary Office in Bern, Switzerland (permit number: BE16/16).

### Statistical Analyses

All statistical analyses were performed using IBM™ SPSS Statistics (version 23), except for the judgment bias task data, which were analyzed using R (version 3.3.2). For parametric models run in SPSS, assumptions of normally distributed errors and homogeneity of variance were examined graphically and, based on these inspections, no transformations of data were needed. Strains of mice were analyzed separately. In SPSS, batch was included in all analyses as a fixed effect—no significant differences were observed and this predictor is not discussed further. *P*-values less than 0.05 were considered statistically significant for all analyses and are presented as actual values rounded to three decimal places. Raw data for all outcome measures will be made available upon request.

Home-cage and stereotypic behavior was analyzed using Kruskal-Wallis independent sample tests due to the high degree of skewness observed in the data. Data was summed across all observation intervals and expressed as proportions. Significant effects were probed using Kruskal-Wallis pairwise comparisons with a Dunn–Bonferroni correction.

Measures of anxiety—elevated plus–maze and open field behavior—were analyzed using the MIXED procedure. Housing condition and, for the open field only, day of testing, were treated as categorical fixed effects. Mouse nested in cage was added as a random effect. Significant effects were probed with Bonferroni corrected pairwise comparisons.

Food and water intake and body weight were analyzed using the MIXED procedure, with housing condition treated as a categorical fixed effect, and week as a continuous covariate. For body weight, mouse nested in cage was added as a random effect. Significant effects were probed with Bonferroni corrected linear contrasts.

One of our measures of stress physiology—fecal glucocorticoid metabolite concentrations—was analyzed using the MIXED procedure. Housing condition and time point of assessment were treated as categorical fixed effects. Mouse nested in cage was added as a random effect. Significant effects were probed with Bonferroni corrected pairwise comparisons. Adrenal mass between housing conditions was analyzed using Kruskal-Wallis independent sample tests due to the high degree of skewness observed in the data. Significant effects were probed using Kruskal-Wallis pairwise comparison with a Dunn–Bonferroni correction.

Measures of brain function—recurrent perseveration and patterned responding in a two-choice guessing task—were analyzed using the MIXED procedure. Housing condition was treated as a categorical fixed effect. Significant effects were probed with Bonferroni corrected pairwise comparisons.

Our measure of emotional valence—responding in a judgment bias task—was analyzed using the function glmer of the package lme4 (“family:” binomial, including the “logit” link function). Housing condition, trial type, and their two–way interaction were fixed effects, while trial type nested in session per test day nested in test day, nested in animal ID nested in batch was used as a random effect.

To assess variation between housing conditions, the coefficient of variation (CV), the ratio of the standard deviation to the mean, for each outcome was calculated from the raw data with the exception of home–cage and stereotypic behavior, guessing task and judgment bias.

## Results

### Engagement With the Housing Environment

**Figure 2:**
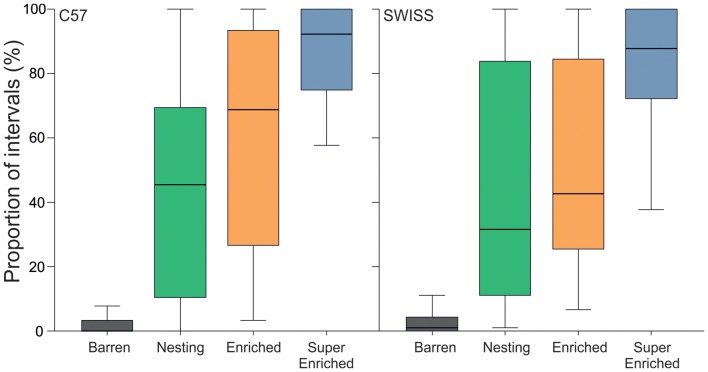
Proportion of intervals across observations (median and IQR) where mice were engaged with the housing environment.

Engagement with the housing environment was defined as the mouse being either in contact with an enrichment item (inside, on, or under) or actively manipulating an enrichment item (N, E, SE) or bedding (all groups). The degree of cage enrichment strongly affected engagement with the housing environment in both strains (C57: *H* = 116.20, *p* < 0.001; SWISS: *H* = 114.80, *p* < 0.001, Figure 2). *Post hoc* comparisons indicated that in both strains of mice, animals housed in the barren condition were less often observed to be engaged with the housing environment compared to all other groups. Additionally, in both strains, animals housed in the super enriched condition were more often observed engaged with the housing environment compared to animals housed in the nesting condition, and in SWISS mice only, compared to the enriched condition. In the SE housing condition, the mice were engaged with enrichments on average more than 85% of the observed time.

### Stereotypic Behavior

**Figure 3:**
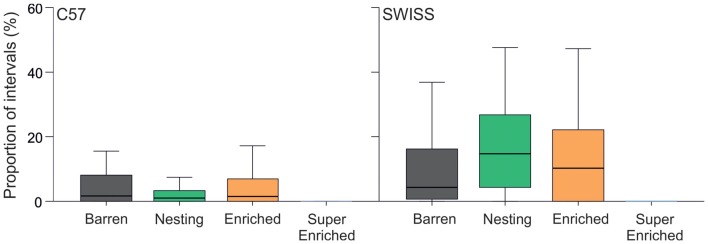
Proportion of intervals across observations (median and IQR) where mice were engaged in stereotypic behavior in relation to housing condition. Note, the *y*-axis is truncated to 60% from 100% to aid with visual clarity, given the low levels of stereotypic behavior.

The degree of cage enrichment had a strong effect on the expression of stereotypic behavior in the home–cage in both strains (C57: *H* = 34.21, *p* < 0.001; SWISS: *H* = 21.77, *p* < 0.001; Figure 3). This was mainly due to greatly reduced stereotypy levels in mice in SE cages compared to mice from all other treatment groups, while there were no consistent differences between mice in B, N and E cages.

The degree of cage enrichment also had effects on specific forms of stereotypies, but these varied with the strain of mouse. The expression of bar-mouthing was affected by cage enrichment in both strains (C57: *H* = 21.82, *p* < 0.001; SWISS: *H* = 16.48, *p* < 0.001; Supplementary Figure S7). However, circling on the cage lid was affected by cage enrichment in C57 mice only (C57: *circling: H =* 20.05, *p* < 0.001), while back-flipping, route-tracing on the cage lid and twirling were affected by cage enrichment in SWISS mice only (SWISS: *back–flipping*: *H* = 20.68; *p* < 0.001;* twirling*: *H* = 13.81; *p* < 0.001; *route–tracing on the lid*: *H* = 11.00; *p* = 0.012; Supplementary Figure S7). These effects were mostly due to higher levels of stereotypies observed in B and N cages compared to E and SE cages, although bar–mouthing in C57 mice was higher in E cages compared to all other cages. However, there were large individual differences in both the form and levels of stereotypy performance, and many 0 values for most forms of stereotypies, which precluded further analysis of treatment effects on specific forms of stereotypies.

### Other Home–Cage Behavior

**Figure 4:**
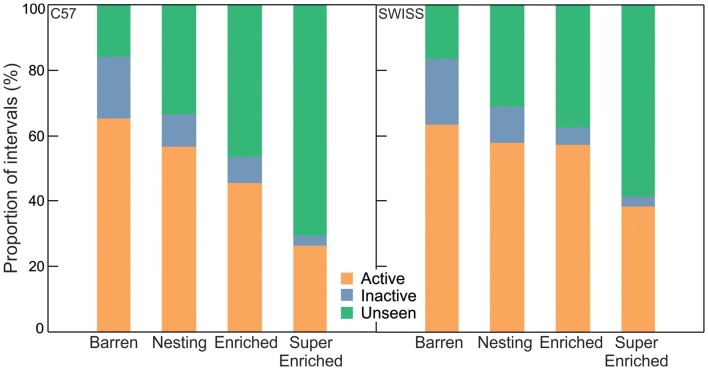
Proportion of intervals engaged in active, inactive and unseen behavior in relation to cage enrichment.

In both strains, variation in home–cage behavior depending on the degree of cage enrichment was mostly determined by the extent of unseen behavior (C57; *active*: *H* = 43.28, *p* < 0.001; *inactive*: *H* = 53.05; *p* = 0.001; *unseen*: *H* = 98.14, *p* = 0.001; SWISS; *active*: *H* = 30.48, *p* < 0.001; *inactive*: *H* = 75.42; *p* < 0.001; *unseen*: *H* = 94.78, *p* < 0.001, Figure 4). Behavior was recorded as unseen when enrichments obscured the view of the mice such that the specific pattern of behavior could not be identified unambiguously. That both the relative amount of seen active and inactive behavior decreased with increasing enrichment indicates that engagement with enrichment affected both active and inactive components of behavior. That seen inactive behavior decreased more than seen active behavior further indicates that mice have a stronger preference for shelter when inactive.

**Figure 5:**
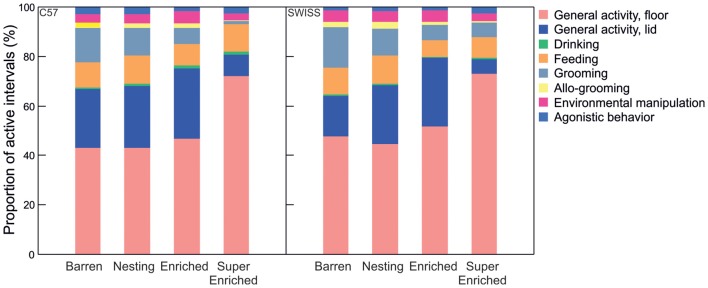
Proportion of intervals engaged in different forms of active behavior in relation to housing condition.

Comparing variation among specific patterns of seen active behavior indicated that engagement with enrichments affected specific patterns of active behavior differently. For example, the proportion of grooming decreased with the degree of enrichment across all treatment groups, while the proportion of active behavior on the lid was greatly reduced, and the proportion of active behavior on the floor increased, in SE mice compared to mice from all other groups (Figure 5).

### Anxiety in the Elevated Plus–Maze Test and Open Field Test

**Figure 6:**
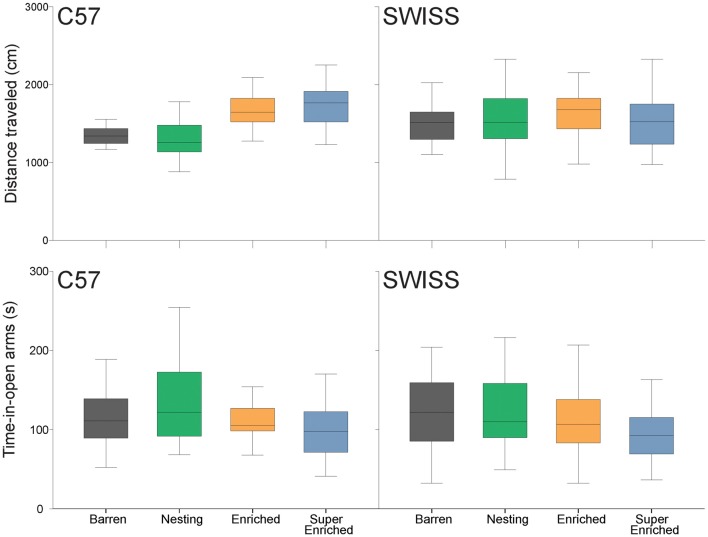
Variation in distance traveled and time-in-open arms (median and IQR) in relation to housing condition.

Behavior in the elevated plus–maze test varied with the type of housing condition in C57 but not SWISS mice. However, this effect was only observed with respect to the total distance traveled but not time-in-open arms (*distance traveled*: C57: *F*_(3,28)_ = 6.53, *p* = 0.002; SWISS: *F*_(3,28)_ = 0.09, *p* = 0.967; *time-in-open arms* C57: *F*_(3,28)_ = 0.95, *p* = 0.080; SWISS: *F*_(3,28)_ = 2.33, *p* = 0.420; Figure 6). Closer inspection of the data indicated that C57 mice housed in E and SE cages traveled longer distances than those housed in B and N cages, while there were no consistent effects with respect to time-in-open arms.

A secondary *post hoc* analysis evaluating time spent in the center of the maze yielded no differences between our treatment groups (C57: *F*_3,91_ = 2.29, *p* = 0.083; SWISS: *F*_3,92_ = 0.46, *p* = 0.709).

**Figure 7:**
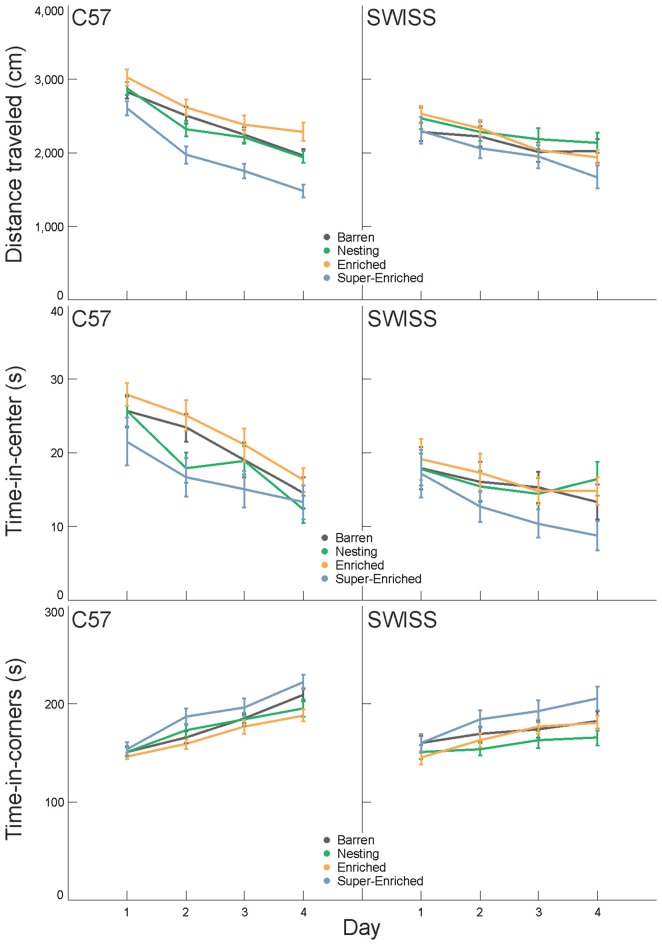
Variation in distance traveled, time-in-center and time-in-corners (mean ± SE) in relation to housing condition and day of testing.

Behavior in the open field varied with the type of housing condition in C57 but not SWISS mice. This effect was observed with respect to both total distance traveled and time in corners, but not time in the center (*distance traveled* C57: *F*_(3,90)_ = 8.18, *p* < 0.001; SWISS: *F*_(3,90)_ = 2.106, *p* = 0.105; *time-in-center* C57: *F*_(3,89)_ = 0.11, *p* = 0.234; SWISS: *F*_(3,88)_ = 1.45, *p* = 0.957;* time-in-corners* C57: *F*_(3,89)_ = 3.43, *p* = 0.021; SWISS: *F*_(3,89)_ = 2.25, *p* = 0.088; Figure 7). In both strains, behavior in the open field also varied across days of testing (*distance traveled* C57: *F*_(3,144)_ = 100.32, *p* < 0.001; SWISS: *F*_(3,171)_ = 9.44, *p* < 0.001; *time-in-center* C57: *F*_(3,169)_ = 28.65, *p* < 0.001; SWISS: *F*_(3,173)_ = 7.84, *p* < 0.001;* time-in-corners* C57: *F*_(3,173)_ = 68.04, *p* < 0.001; SWISS: *F*_(3,174)_ = 21.95, *p* < 0.001; Figure 7). However, there was no interaction between the type of housing condition and day of testing (*distance traveled* C57: *F*_(9,144)_ = 1.64, *p* = 0.109; SWISS: *F*_(9,171)_ = 1.84, *p =* 0.064; *time-in-center* C57: *F*_(9,169)_ = 0.63, *p* = 0.773; SWISS: *F*_(9,173)_ = 0.72, *p* = 0.687;* time-in-corners* C57: *F*_(9,173)_ = 1.62, *p* = 0.114; SWISS: *F*_(9,174)_ = 1.59, *p* = 0.121; Figure 7). Closer inspection of the data indicated that both distance traveled and time in the center decreased while time in the corner increased across the 4 days of testing. Furthermore, C57 mice housed in SE cages traveled shorter distances compared to all other groups.

### Growth: Body Weight and Food and Water Intake Per Mouse

In both strains and across all housing conditions, food intake was positively correlated with both water intake and body weight (averaged at the cage level)—except for C57 mice housed in E and SE groups (Supplementary Table S6). In C57 mice only, water intake was positively correlated with body weight (averaged at the cage level) across all housing conditions—a similar relationship was observed only in SWISS mice housed in the SE condition (Supplementary Table S6).

**Figure 8:**
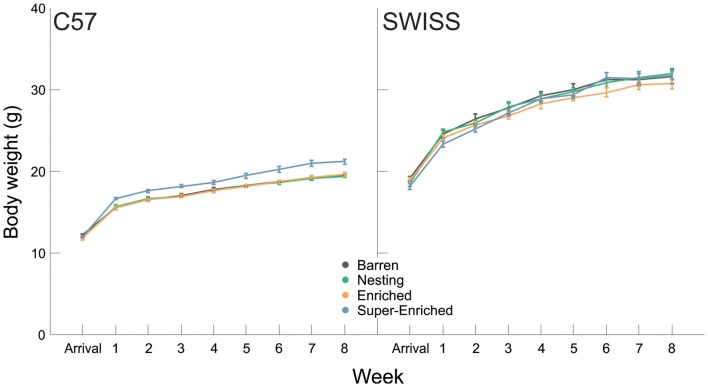
Variation in body weight (mean ± SE) in relation to housing condition and week.

Body weight at arrival did not vary between housing conditions in both strains (C57: *F*_(3,87)_ = 0.79, *p* = 0.504; SWISS: *F*_(3,88)_ = 1.55, *p* = 0.208). Body weight increased with age in both strains (C57: *F*_(8,333)_ = 725.88, *p* < 0.001; SWISS: *F*_(8,348)_ = 495.52, *p* < 0.001; Figure 8). Furthermore, body weight varied with the type of housing condition in C57, but not SWISS mice (C57: *F*_(3,99)_ = 13.04, *p* < 0.001; SWISS: *F*_(3,88)_ = 0.70, *p* = 0.543), and there was an interaction between the type of housing condition and week in C57, but not SWISS mice (C57: *F*_(24,333)_ = 3.77, *p* < 0.000; SWISS: *F*_(24,348)_ = 1.51, *p* = 0.060; Figure 8). *Post hoc* comparisons indicated that in C57 mice only, mice housed in the SE condition were heavier than mice of all other housing conditions. This difference emerged 1 week after arrival and persisted for the duration of the experiment.

**Figure 9:**
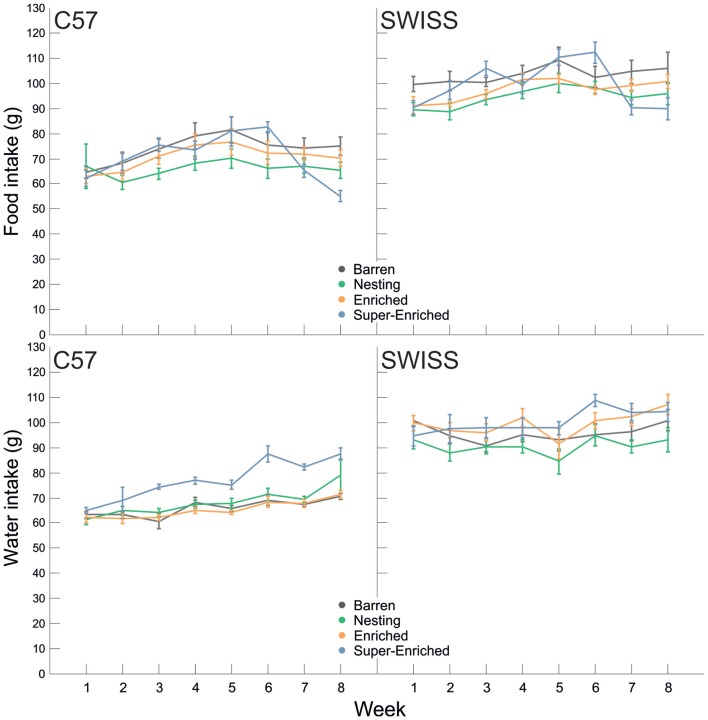
Variation in food and water intake (Mean ± SE) in relation to housing condition and week.

In both strains, there was no main effect of the type of housing condition on food intake (C57: *F*_(3,28)_ = 1.19, *p* = 0.332; SWISS: *F*_(3,28)_ = 2.88, *p* = 0.054), but food intake varied by week (C57: *F*_(7,66)_ = 11.16, *p* < 0.001; SWISS: *F*_(7,107)_ = 9.27, *p* < 0.001). Furthermore, there was an interaction between the type of housing condition and week in both strains *(*C57: *F*_(21,66)_ = 3.76, *p* < 0.001; SWISS: *F*_(21,107_ = 4.27, *p* < 0.001; Figure 9). Closer inspection of the data indicated that food intake initially increased in both strains and all treatment groups, but flattened or decreased with age, whereby these changes in the time course of food intake differed between treatment groups. Thus, there was a relative decrease in food intake with increasing degree of enrichment at later time points.

Water intake increased with age (C57: *F*_(7,118)_ = 5.2, *p* < 0.001; SWISS: *F*_(7,120)_ = 8.66, *p* < 0.001) and there was a main effect of the type of housing condition on water intake (C57: *F*_(3,28)_ = 15.37, *p* < 0.001; SWISS: *F*_(3,28)_ = 3.01, *p* = 0.047). Furthermore, in SWSS, but not C57 mice, water intake also varied depending on the interaction between the type of housing condition and week (C57: F_(21,118)_ = 0.880, *p* = 0.606; SWISS: *F*_(21,120)_ = 2.14, *p* = 0.006; Figure 9). *Post hoc* comparisons indicated, in both strains of mice, that average water intake was higher in the SE cages. In C57 mice, water intake was higher in the SE group from week 3 compared to all other groups. In SWISS mice, water intake was higher in the SE group on week 6 compared to B and E groups.

### Endocrine Stress Responses: Fecal Glucocorticoid Metabolites and Adrenal Weight

**Figure 10:**
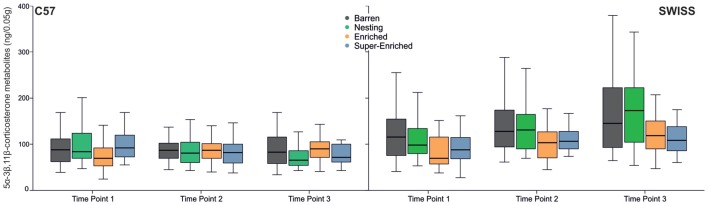
Variation in glucocorticoid metabolite concentrations (median and IQR) in relation to housing condition and time point of measurement.

Glucocorticoid metabolite concentration increased across the three time points in SWISS mice, but not in C57 mice (C57: *F*_(2,125)_ = 0.52, *p* = 0.596; SWISS: *F*_(2,142)_ = 20.67, *p* < 0.000; Figure 10). Furthermore, there was an effect of the type of housing condition in SWISS, but not C57 mice (C57: *F*_(3,91)_ = 0.18, *p* = 0.910; SWISS: *F*_(3,89)_ = 4.38, *p* = 0.006). *Post hoc* comparisons indicated that glucocorticoid metabolite concentration was, on average, higher in mice housed in B and N cages compared to mice housed in E and SE cages across all three time-points; but only in SWISS mice. There was also an interaction between type of housing condition and time point in C57 mice, but not SWISS mice (C57: *F*_(6,126)_ = 2.38, *p* = 0.032; SWISS: *F*_(6,142)_ = 0.64, *p* = 0.687), but *post hoc* analyses yielded no consistent effects.

**Figure 11:**
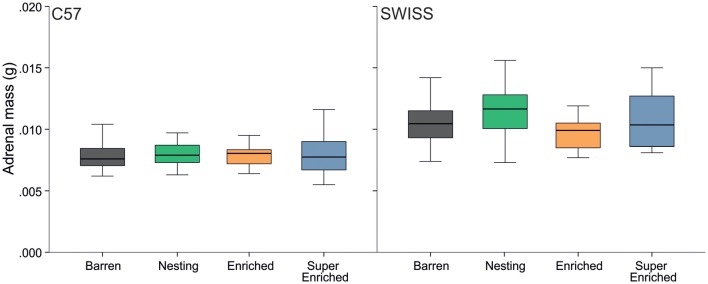
Variation in adrenal mass (median and IQR) in relation to housing condition.

Variation in adrenal weight partly reflected the effects found in glucocorticoid metabolite concentrations. Thus, the type of housing condition affected adrenal weight in SWISS mice, but not C57 mice (C57: *H =* 2.95, *p* = 0.400; SWISS:* H* = 13.12, *p* = 0.004; Figure 11). However, *post hoc* comparisons indicated a difference in adrenal weight only between mice from N cages compared to mice from E cages.

### Brain Function: Recurrent Perseveration in a Two–Choice Guessing Task

**Figure 12:**
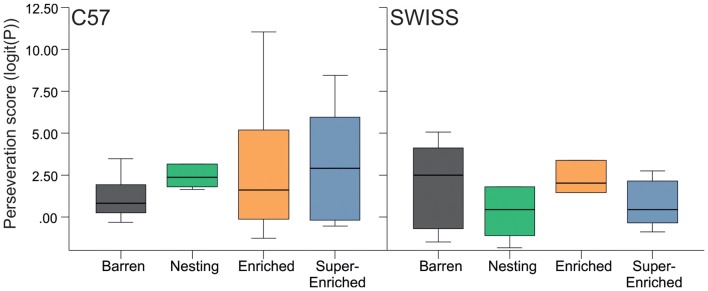
Variation in perseveration score (median and IQR) in relation to housing condition.

Recurrent perseveration was not affected by housing condition, stereotypy level, or by the interaction between the type of housing condition and stereotypy level (C57; *housing condition*: *F*_(3,16)_ = 0.57, *p* = 0.645; *stereotypy level*: *F*_(1,16)_ = 0.38, *p* = 0.850; *housing condition × stereotypy level*: *F*_(3,16)_ = 0.43, *p* = 0.732; SWISS; *housing condition*: *F*_(3,16)_ = 1.09, *p* = 0.384; *stereotypy level*: *F*_(1,16)_ = 0.38, *p* = 0.549; *housing condition* × *stereotypy level*: *F*_(3,16)_ = 0.71, *p* = 0.558; Figure 12).

**Figure 13:**
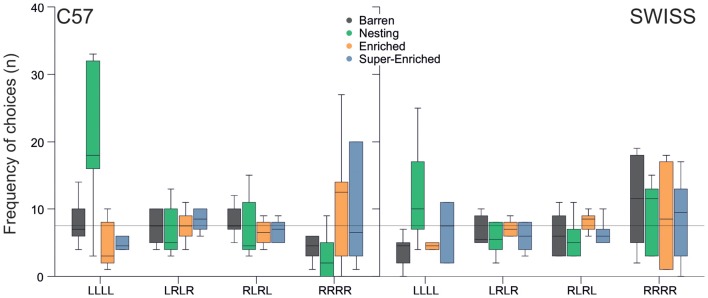
Variation in repetitions and alternations in relation to housing condition (median and IQR). The line represents the expected frequency of choices given a random search strategy.

Response patterns in the guessing task varied with the type of housing condition in C57 mice, but not SWISS mice (C57; *F*_(9,80)_ = 4.31, *p* < 0.001; SWISS; *F*_(3,16)_ = 1.22, *p* = 0.295; Figure 13). Closer inspection of the data indicated that C57 mice housed in N cages made more pure repetitions compared to all other groups, albeit to one side only (LLLL).

### Emotional State: Judgment Bias in a Spatial Go/No–Go Task

All C57 mice reached the learning criterion for testing, whereas two SWISS mice were excluded during the Go/No–go Discrimination stage (see Hintze et al., 2018 for further methodological details). Total training duration for all stages (Habituation, Shaping for Trial Initiation, Left–Right Discrimination, Go/No–go Discrimination) was 14.20 ± 1.63 sessions for C57 mice and 15.50 ± 1.79 sessions for those SWISS mice that reached the test criterion.

**Figure 14:**
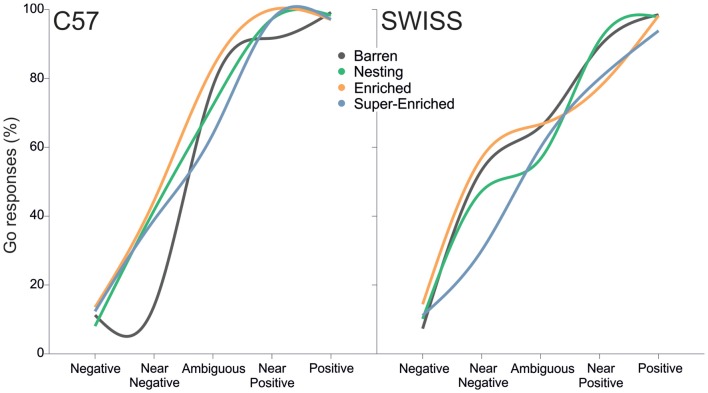
Fitted regression lines showing go responses in relation to housing condition and strain.

During testing, the animals’ decision as to whether or not to go when confronted with the different trial types varied as an interaction between the type of housing condition and trial type for both C57 and SWISS mice (C57: $\chi_{3}^{2}$ = 13.46, *p* = 0.004; SWISS: $\chi_{3}^{2}$ = 22.97, *p* < 0.001; Figure 14).

Closer inspection of the data revealed no consistent differences in response patterns depending on the type of housing condition. In both strains, the strongest treatment effect was observed in near-negative (NN) trials, as indicated by fewer go responses in mice from B cages in C57 mice, and in mice from SE cages in SWISS mice, compared to mice from all other treatment groups. However, NN trials were also associated with the largest variability in the number of go responses.

### Variability in the Measured Outcome Variables

To assess potential effects of the type of housing condition on the variability of experimental results, we calculated the CV for all measured outcome variables, with the exception of home–cage and stereotypic behavior, and for judgment bias and guessing task data where only a subset of animals were tested (*n* = 6 per strain per housing condition). The CV is the ratio of the standard deviation to the mean, which yields a dimensionless, standardized measure of dispersion. It thus allowed us to compare variation estimates directly between the different outcomes measured in this experiment.

**Figure 15:**
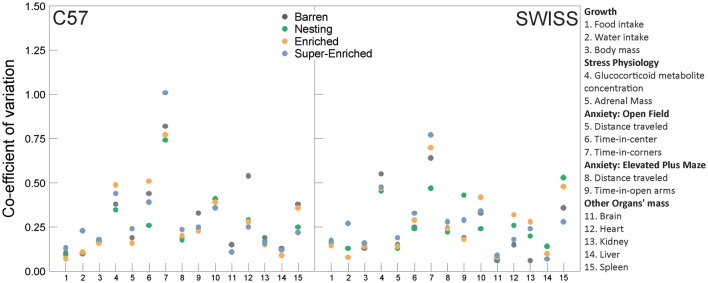
Co-efficient of variation estimates by outcome measure, housing condition and strain.

CVs varied greatly depending on the measured outcome variable (Figure 15, Supplementary Table S7). Most CVs were relatively small and there were no consistent relationships between cage enrichment and CV across the range of outcome variables assessed in this study.

## Discussion

To study the effects of environmental enrichment on measures of animal welfare in mice, we systematically varied housing conditions across four levels of enrichment and measured animal welfare in a multi-faceted way. Overall, the greatest benefit to welfare was observed in animals housed with the most extensive enrichment, the SE housing condition—stereotypic behavior, some behavioral measures of anxiety, growth and stress physiology, all varied in a manner consistent with improved animal welfare. We also assessed the effect of the different housing conditions on variation in outcome measures. Similarly to other studies (Augustsson et al., 2003; Wolfer et al., 2004; Baumans et al., 2010; van de Weerd et al., 2010), we found no evidence that environmental enrichment increased variation in experimental results, for the outcome measures assessed here.

When evaluating whether and how animals engaged with the enrichment items and evaluating the relation to welfare, substantial differences were noted—animals were more often observed to be in, on or under items, and manipulated items as the degree of enrichment increased. One possible explanation for this difference may be that there is a higher probability for contact with different items, simply by chance, as the number of items increased and the “free” floor area decreased. If this explanation were true, we would predict that despite higher levels of engagement with the housing environment, animal welfare would not be improved. This explanation may apply to N and E groups—even though engagement with enrichment increased with the degree of enrichment, no consistent improvements in measures of welfare were observed in comparison to the B housing condition. For example, the expression of stereotypic behavior did not vary in a systematic way between B, N and E housing groups even though engagement with enrichments increased with increasing degrees of enrichment in these groups. However, this does not apply to the SE housing condition. Although the SE condition was the most enriched, it also offered the most “free” floor and lid space. Furthermore, in the SE housing condition, virtually no stereotypic behavior was observed—suggesting improved welfare in this group (Mason and Latham, 2004). It is possible that the prevalence of stereotypic behavior was underestimated in the SE group, given that the highest levels of unseen behavior were also observed in this group. We find this explanation unlikely given that bar–mouthing, the most commonly reported form of stereotypy in laboratory mice (Würbel, 2006), was observable and recorded since the bars of the cage were not covered by enrichment items. Animals in the SE group displayed extremely low levels of bar–mouthing (on average <1%). It is similarly unlikely that mice were performing any of the other forms of stereotypy categorized here when unseen, given the amount of space that is necessary for the performance of these behaviors; most of the unseen behavior was recorded when mice were under or in enrichment items. Therefore, this difference in stereotypy performance, with respect to housing condition, likely reflects the true incidence of behavioral expression. Underestimation of stereotypic behavior, when animals were unseen, in B, N and E cages is also unlikely given that unseen behavior in these cages was primarily scored when animals were either in the nest or in a huddle and where one mouse visibly blocked another. Neither of these two scenarios is likely to have masked the display of stereotypic behavior.

When assessing differences in time budget, some differences were observed—for example, the proportion of grooming and of active behavior on the lid decreased with the degree of enrichment across all treatment groups. In contrast to stereotypic behavior, however, other components of the animals’ time budget categorized here could occur when an animal was in or under enrichment items. For example, we frequently observed nesting material shaking in a rhythmic pattern, indicative of grooming or allo-grooming behavior; this behavior was coded as unseen, given that the animals were not visible. Therefore, it is quite likely that our time–budget assessment underestimates the prevalence of specific behaviors and these data should therefore be interpreted with caution. On the other hand, as our data indicated that grooming was often performed out of sight, it may also suggest that mice prefer to perform grooming in a shelter, if a shelter is available.

Differences in engagement with enrichment items were also associated with improvements in animal welfare, albeit in a strain dependent way. C57 mice housed with a greater degree of enrichment, i.e., E and SE groups, were more exploratory, and therefore less anxious, in the elevated plus–maze. In the open field, C57 mice housed in the SE groups displayed a classic habituation response to the novelty of the arena across days of testing—on average lower levels of exploration and more time spent inactive in the corner of the arena. Thus, in general, C57 mice housed in SE cages displayed an anxiolytic profile in comparison to the other housing conditions.

In terms of our secondary outcome measures, some concordant differences were observed. For example, when comparing differences in stress physiology, a consistent difference was observed, but only in SWISS mice—animals housed in E and SE groups had lower levels of glucocorticoid metabolites in feces across all three time points of measurement compared to B and N groups. This result suggested that SWISS mice housed in E and SE conditions experienced lower levels of chronic stress. Our other measure of stress physiology, adrenal weight, varied differently with housing condition—SWISS mice housed in E groups had smaller adrenals compared to those housed in the N groups. Both adrenal weight and glucocorticoid metabolites in feces have been used previously as indicators of experienced chronic stress in mice (Tsai et al., 2002; Akre et al., 2011; Gurfein et al., 2014; Bailoo et al., 2018), albeit with different degrees of sensitivity relative to housing conditions. For example, one study has found that animals housed in enriched environments tended to have smaller adrenals; although this difference was not statistically significant (Tsai et al., 2002). In contrast, studies that measured corticosterone secretion in feces have consistently found that enriched housing conditions are associated with decreased levels of circulating glucocorticoids (Akre et al., 2011; Gurfein et al., 2014). Therefore, the discrepancy in the pattern of differences between fecal glucocorticoids and adrenal weight with respect to housing condition may simply reflect differences in measurement sensitivity.

Across time, food intake initially did not vary by housing condition—it did however vary by time. Food intake initially increased, peaking at around 7–8 weeks of age (puberty/early adulthood) and then either flattened or decreased with age. In contrast, water intake increased across time in the SE condition in both strains, but did not vary between the other housing conditions. In C57 mice only, animals housed in the SE condition weighed more than animals in all other groups. Notably, food intake and body weight (averaged at the cage level) were uncorrelated in C57 mice housed in E and SE groups. Taken together, these patterns of differences with respect to growth, most likely reflect variation in metabolic need. Importantly, as ambient temperatures in mouse facilities are kept below the thermoneutral zone of mice, increasing the risk of cold stress (Gordon, 1985, 1993; Johnson et al., 2017), increased opportunity for structural complexity, and in particular for the construction of elaborate nests, may act as a buffer to cold stress—resulting in heavier animals. However, we were unable to examine this hypothesis further, as we did not measure temperatures inside the cages (micro-climate). Food and water intake are tightly linked to metabolism and are, in general, positively correlated (Gordon, 1993; Gordon et al., 1998). However, in relative terms, water intake in mice is an inelastic requirement—in the absence of water, mice quickly dehydrate (Harkness et al., 2013). Increased water intake in the SE housing condition is therefore most likely reflective of a difference in metabolic need prompted by increased activity and possibly reflecting increased engagement with the environment.

Animals housed in the different housing conditions did not vary in our measures of brain function, recurrent perseveration and patterned responding in a two-choice guessing task; at least not in the predicted way. Specifically, animals housed in barren environments have been reported to display a higher incidence of stereotypic behavior and, in turn, higher levels of perseverative behavior and patterned responding (Garner et al., 2011). In this experiment, no association was found between housing condition, stereotypy level and recurrent perseveration. This lack of association is, however, in line with more recent studies using this experimental paradigm (Latham and Mason, 2010; Gross et al., 2011a, 2012; Novak et al., 2016; Bailoo et al., 2018); the levels of stereotypy observed here were similar to those in the study reporting a relationship (Garner et al., 2011). These findings therefore indicate that either this test paradigm does not measure recurrent perseveration reliably or that cage stereotypies in these mice do not reflect behavioral disinhibition as measured by recurrent perseveration. A difference in patterned responding was observed with respect to housing condition, in C57 mice only—animals housed in the nesting condition made more repetitive choices, compared to all other groups; albeit to one side (LLLL). Previous studies have indicated that C57 mice tend to show more alternations (LRLR or RLRL) than repetitions (LLLL or RRRR; Bailoo et al., 2018) and that enriched housing reduces the number of repetitive choices compared to barren housing (Gross et al., 2011a). The series of paradoxical results across studies suggests that these may be study specific idiosyncrasies of unknown etiology.

Studies investigating the effect of transfer from standard housing conditions to enriched environments on judgment biases in rats have reported a shift in judgment biases from more “pessimistic” to more “optimistic” responses (Brydges et al., 2011; Richter et al., 2012). In the present experiment, the overall response pattern, a monotonic graded response, was observed in mice from both strains and across all four housing conditions—confirming that the internal consistency criteria of this test were met (for further details, see Hintze et al., 2018). However, the only variations depending on housing conditions were that C57 mice housed in the B group and SWISS mice kept in the SE group showed a lower number of go responses when confronted with near negative trials compared to the other housing conditions. These findings may indicate either that this test was not sensitive enough to detect variation in animal welfare or that the observed differences in animal welfare were not associated with differences in emotional valence. However, judgment bias was assessed at the end of a series of other tests and after extensive training, with daily removal from the home cage and handling for testing, which may have masked treatment effects. Without further study, this explanation remains essentially speculative. Therefore, these results—the first report of judgment biases in mice as a consequence of environmental supplementation—require replication and further study.

When comparing variation across our outcome measures in relation to housing conditions, no systematic patterns of differences were found. These results add to a growing body of evidence against the often heard, yet unsubstantiated, concerns that environmental enrichment would increase variation in outcome measures, thereby inducing a need for larger sample sizes to detect treatment effects (Augustsson et al., 2003; Wolfer et al., 2004; Baumans et al., 2010; van de Weerd et al., 2010).

Overall, we found that the mice readily used the enrichments when available, and that engagement with enrichment increased with increasing degree of enrichment—indicating that the enrichments offered the animals choices, which they integrated in the expression of their behavior. However, we also found that with the exception of extensive enrichment in the SE condition, variation in enrichment did not produce consistent variation in our measures of welfare. According to a large body of literature, there is no doubt that nesting material is beneficial for laboratory mice in many ways (Bult and Lynch, 1997; Sherwin, 1997; van de Weerd et al., 1997; Olsson and Dahlborn, 2002; Smith and Corrow, 2005; Gaskill et al., 2009, 2011, 2012; Gross et al., 2011b). Previous studies also found that nesting material alone attenuates stereotypic behavior (Gross et al., 2012). However, the lack of consistent differences between mice housed in B, N and E conditions together with the substantial differences between these mice and those housed in SE conditions suggested that considerably more extensive enrichment strategies may be needed to achieve substantial improvements in welfare. From the present results, we do not know whether it was the larger space, the frequency of bedding changes, the more complex environment, the types of enrichments, or the novelty that contributed most to the beneficial effect of the SE conditions as all of these factors were confounded. We therefore suggest that further studies under more extensive conditions are needed to inform decisions on minimal requirements for the housing of laboratory mice. Such an approach may allow for the identification of relevant resources or combinations of resources necessary for improving the welfare of laboratory housed rodents. It may also lend insight into relevant mental or cognitive capacities underlying species-typical behaviors (e.g., learning, memory, spatial navigation)—permitting for the development of innovative housing solutions that recapitulate the use of these capacities in the laboratory (for review, see Bennett et al., 2018; and for examples of evaluation and application, Dutton et al., 2018). In the 70s and 80s, such an approach was pursued in a range of farm animals (e.g., laying hens: Fölsch et al., 1983; rabbits: Stauffacher, 1992; pigs: Stolba and Wood-Gush, 1984), resulting in welfare-friendly prototypes of housing conditions, from which practicable solutions were further developed for specific contexts of animal use (e.g., laboratory rabbits: Stauffacher, 1994).

The present results also highlight the difficulty in making general recommendations for improving the housing environment of laboratory mice. For example, even though the mice engaged more with the environment as the degree of supplementation increased, specific improvements in measures of welfare varied in a strain specific way. Furthermore, we only studied females and the needs of male mice may differ from those of female mice due to their higher propensity of escalating aggression (Kappel et al., 2017). Thus, we need to take into consideration that there may be no single solution to meet the needs of all strains of mice and both sexes, and enrichment strategies may always have to be adjusted to the specific strain and sex being used.

## Author Contributions

HW: funding acquisition and resources. JB, EM and HW: conceptualization. JDB and EM: methodology. JB: project administration, formal analysis and writing–original draft. JB, EM, MB-S, JV, SH, CB, KH, CG and RP: investigation. JB EM MB-S, JV, SH, KH, CG, RP, BV and HW: writing–review and editing.

## Conflict of Interest Statement

The authors declare that the research was conducted in the absence of any commercial or financial relationships that could be construed as a potential conflict of interest.
