# Supplementary material for: Effects of Cage Enrichment on Behavior, Welfare and Outcome Variability in Female Mice
Source: Front Behav Neurosci. 2018 Oct 26;12:232. doi: 10.3389/fnbeh.2018.00232 (PMC6212514; doi:10.3389/fnbeh.2018.00232)

Effects of cage enrichment on behavior, welfare, and outcome variability in female mice

Jeremy D. Bailoo[1], Eimear Murphy[1], Maria Boada-Saña[1], Justin A. Varholick[1], Sara Hintze[2], Caroline Baussière[1], Kerstin C. Hahn[3], Christine Göpfert[3], Rupert Palme[4], Bernhard Voelkl[1] and Hanno Würbel[1]

[1]Division of Animal Welfare, Veterinary Public Health Institute, University of Bern, CH

[2]Division of Livestock Sciences, Department of Sustainable Agricultural Systems, University of Natural Resources and Life Sciences Vienna (BOKU), Vienna, AT

[3]Institute for Animal Pathology, University of Bern, CH

[4]Department of Biomedical Sciences, University of Veterinary Medicine Vienna, AT

Supplementary Information:
Number of pages: 10
Number of figures: 7
Number of tables: 7

Supplementary Methods

Husbandry procedures

Animals were delivered on a Tuesday, and because our weekly husbandry procedures occur on a Friday, the first set of routine husbandry procedures was performed 10 days after arrival to the lab, with one week intervals thereafter. All husbandry procedures occurred in the dark phase under red light. During husbandry, Type 3 cages, water bottles, and nesting material and enrichment items contained in the Type 3 cages, where applicable, were changed. Additionally, in the large SE enclosure, the food, water, hammock, paper tunnel, paper shelter, running wheel (from week 4 onward), and the raised platform (including bedding) were changed weekly while the bedding on the floor of the cage was changed every 5 weeks.

In the first 10 days of the experiment, animals in the SE group were housed in the Type 3 cage without access to the large SE enclosure, in order to facilitate monitoring of these animals and to ensure that an association with food and water being present in this cage was formed. This association was necessary because animals in the SE cage were removed for husbandry and testing procedures by shuttling the animals into the Type 3 cage, closing access to the large SE enclosure, and then taking the animals in the Type 3 cage to the testing room (c.f., Supplementary Fig. 1). Most animals learned this association and came readily to the Type 3 cage by the onset of testing.

Handling procedure

Each cage of animals was handled by one of three experimenters (EM, MB-S, JDB) on a rotating schedule such that no cage of animals was handled consecutively by the same experimenter. Handling consisted of gently scooping the animal in the palm of the hand and allowing it to explore for approximately ten seconds before returning it to the cage. A cage-mate was then scooped, and the procedure repeated. The first 5 handling sessions lasted five minutes, and each experimenter ensured that each animal was handled at least five times in

each of these sessions. Thereafter, handling sessions lasted three minute, where each animal was handled at least thrice. Throughout the experiment animals were never handled by the tail, with the exception of during restraint for ear–tattooing or for specific health monitoring.

Supplementary Table 1. Description of the four housing conditions used in this study.

Housing Condition	Cage Type	Floor Area (cm^2)	Height (cm)	Bedding Depth (cm)	Enrichment
Barren	Makrolon Type 3	820	15.5	1.5	None
Nesting	Makrolon Type 3	820	15.5	1.5	Nesting material
Enriched	Makrolon Type 3	820	21.5	3.0	Nesting material, red plastic tunnel, red plastic shelter
Super-Enriched	Makrolon Type 3	820	21.5	3.0	Nesting material, paper tunnel, paper shelter
	Savic Mickey XL	4000	38.0	6.0	Rotated weekly (see Supplementary Table 3)
	Raised Platform	2000	3.0	1.5	

Supplementary Table 2. Distribution of subjects by treatment, strain and batch

	Condition	Female	# Cages	# Animals
Batch 1	Barren	C57BL/6	4	12
		SWISS	4	12
	Nesting	C57BL/6	4	12
		SWISS	4	12
	Enriched	C57BL/6	4	12
		SWISS	4	12
	Super Enriched	C57BL/6	4	12
		SWISS	4	12
Batch 2	Barren	C57BL/6	4	12
		SWISS	4	12
	Nesting	C57BL/6	4	12
		SWISS	4	12
	Enriched	C57BL/6	4	12
		SWISS	4	12
	Super Enriched	C57BL/6	4	12
		SWISS	4	12
		**Total**	*64*	*192*

Supplementary Table 3: List of items rotated weekly in the Mickey XL cage

Week	Item
1	Kapok seed pod
2	Rope ladder with wodden rungs
3	***Salt lick with herbs****
4	***Running wheel****
5	Kapok seed pod and ***wodden bridge****
6	Tunnel surprise†
7	Tunnel surprise†, trapeze
8	Tunnel surprise†, 3x aspen blocks
9	Tunnel surprise†
10	Tunnel surprise†
11	Tunnel surprise†
12	Tunnel surprise†
13	Tunnel surprise†

**Items remained in the cage, after addition, until the end of the experiment*

†A random mix of dinkel, hay, straw, corncob bedding, mixed nuts & seeds, and Bio-Serv chocolate pellets, placed in paper tunnel with paper tissues.

Supplementary Table 4. Ethogram for the recording of stereotypic behavior. Behavior patterns were considered stereotypic if the same movement sequence was repeated continuously for at least 3 s (bar–mouthing) or at least three times in a row without pauses longer than 3 s between bouts (circling, cage–top twirling, back–flipping, route–tracing).

Category	Name	Definition
General activity	Active	The animal is active throughout the 15s interval
	Inactive	Sitting or lying motionless throughout the 15s interval, occasionally interrupted by brief single twitches lasting no longer than 5s.
Stereotypic behaviour	Bar-mouthing	The mouse holds the cage bar in its diastema and makes a series of sham-biting or open mouth movements along the bar.
	Circling	Repetitive tracing of a circular path either on the cage lid or floor.
	Cage-top twirling	Spinning around the longitudinal body axis while hanging on the cage lid with the forepaws.
	Back-flipping	Backward flip from one cage wall towards the opposite cage wall.
	Route-tracing	Moving along an invariant route on the cage lid or floor.

Supplementary Table 5. Ethogram for the recording of time budget

	Category	Definition
Active Behaviors	**General activity on the floor**	The animal is visible and active and the behaviour being performed does not fall into the below categories or cannot be distinguished.<br>***Modifier:*** In/On Enrichment
	**General activity on the lid**	The animal is visible and active but no limb is in contact with the floor or a piece of enrichment and at least one limb is in contact with the the lid, and the behaviour being performed does not fall into the bellow categories.
	**Drinking**	The animal's head is directed toward the nozzle of the water bottle and the animal's head must be directly in front of the nozzle.
	**Feeding**	The animal's head is directed toward the food hopper (and directly under / in front of the food hopper), manipulation of the food is observed or the snout is brought in contact with food (or bedding).
	**Grooming**	The animal is licking its fur, using its forepaws to manipulate its vibrissae, eyes, face, body or tail or scratching with its limb.
	**Allogrooming**	Grooming behaviour that involves two or more animals. The actor is usually in snout contact with the body of the recipient, with repetitive movement (at least 2) of the snout area over the target area. All mice involved are coded.
	**Environmental Manipulation**	*Behaviours that are subsumed under this category are limited to:*
		Shovelling : This behaviour involves a sequence of two actions - burrowing under the bedding material with the upper half of the body and then resurfacing from the bedding (may shake to remove the substrate). The consequence of this sequence of behaviour is to pile bedding towards a given location. Repetitions of this sequence may be observed.
		Carrying : The animal uses its mouth or picks up with its forepaws to transport nesting material or bedding from one location to the nest.
		Pulling in : The animal is sitting in the nest and is using its forepaws or its mouth to move nesting material or bedding towards itself.
		Digging/Push-digging : The animal uses its forepaws to repeatedly push (forward or backward) nesting material or bedding for the previous 5s.
		Interaction with Enrichment : Movement of the enrichment item is observed while the animal is interacting with it.
	**Agonistic Behavior**	***Mounting/being mounted*** : One mouse climbs unto another one with the forelimbs on its back and the belly in contact with its anogenital region. Both mice are coded.
		Biting : The teeth of one mouse grips the skin of another mouse, and the head is pulled back sharply. This is often directed toward the tail or rump of the recipient.
		Displacement : One mouse comes into contact with another mouse, and the second mouse moves away from its original location. Both mice are coded.
		Chasing : One mouse closely follows another mouse but no other aggressive behaviour is observed. Both mice are coded.
		Stealing : One mouse uses its forepaws or its snout to get in contact with the food of another mouse. The food can be taken or not. Both mice are coded.
	**Inactivity**	The torso and/or head animal is visible and has been immobile for the previous 5s.<br>***Modifier:*** In/On Enrichment
	**Unseen**	The body of the animal is not visible at all or can not be distinguished at the sample point.<br>***Modifier:*** In/On Enrichment
	**Missing**	Animal is missing due to attrition or euthanasia

Supplementary Table 6. Pearson product–moment correlations comparing food and water intake and averaged body weight

Housing Condition	Outcome Measure	C57 Food Intake	C57 Water Intake	C57 Average Body Weight	SWISS Food Intake	SWISS Water Intake	SWISS Average Body Weight
Barren	Food Intake		0.389**	0.346**		0.583**	0.369**
Nesting			0.610**	0.257**		0.403**	0.521**
Enriched			0.096	0.082		0.399**	0290**
Super-Enriched			0.148	-0.005		0.400**	0.256**
Barren	Water Intake			0.591**			0.084
Nesting				0.592**			0.160
Enriched				0.459**			0.091
Super-Enriched				0.771**			0.568**

***Correlation is significant at the 0.01 level (2-tailed).*

Supplementary Table 7. Co-efficient of variation estimates by outcome measure, housing condition and strain.

Category		Outcome Measure	C57				SWISS			
			Barren	*Nesting*	*Enriched*	*Super-Enriched*	*Barren*	*Nesting*	*Enriched*	*Super-Enriched*
Growth		Food intake	0.105	0.072	0.092	0.132	0.160	0.147	0.166	0.173
		Water intake	0.100	0.110	0.110	0.230	0.080	0.080	0.130	0.270
		Body mass	0.180	0.160	0.170	0.180	0.130	0.140	0.140	0.160
Endocrine Stress Response		Glucocorticoid metabolite concentration	0.379	0.488	0.347	0.440	0.551	0.470	0.454	0.476
		Adrenal mass	0.190	0.160	0.160	0.240	0.150	0.140	0.130	0.190
Anxiety	*Open Field*	Distance traveled	0.440	0.510	0.260	0.390	0.250	0.290	0.240	0.330
		Time-in-center	0.820	0.770	0.740	1.010	0.640	0.700	0.470	0.770
		Time-in-corners	0.192	0.201	0.177	0.237	0.242	0.240	0.222	0.281
	Elevated Plus Maze	Distance traveled	0.330	0.230	0.240	0.250	0.190	0.180	0.430	0.290
		Time-in-open arms	0.410	0.390	0.410	0.360	0.330	0.420	0.240	0.340
Other Organs' Mass		Brain	0.150	0.110	0.110	0.110	0.060	0.080	0.070	0.090
		Heart	0.540	0.280	0.290	0.250	0.150	0.320	0.260	0.180
		Kidney	0.190	0.150	0.170	0.160	0.060	0.280	0.200	0.240
		Liver	0.130	0.090	0.120	0.120	0.100	0.100	0.140	0.070
		Spleen	0.380	0.360	0.250	0.220	0.360	0.480	0.530	0.280

Supplementary Figure 1. Layout of housing and testing rooms for one batch (B=Barren, N=Nesting, E=Enriched, SE=Super Enriched).

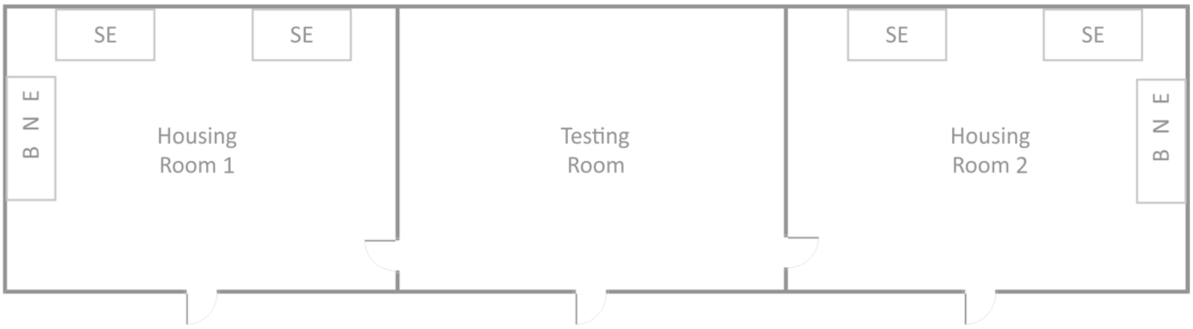

Supplementary Figure 2: Pictures of the different housing conditions used in this experiment. A) Barren; B) Nesting w/ Sizzle Pet; C) Nesting w/Paper Tissues; D) Enriched w/square rat tunnel, square shelter and Sizzle Pet nesting; E) Enriched w/round rat tunnel, round shelter and paper tissues nesting; F) Type 3 cage attached to Super–Enriched Cage, w/ paper tunnel and shelter, Sizzle Pet and Paper Tissue nesting material; G) Front view of Super–Enriched cages; H) Close up view of inside of large (Mickey XL) Super–Enriched cage; and I) Top view of inside of large (Mickey XL) Super–Enriched cage

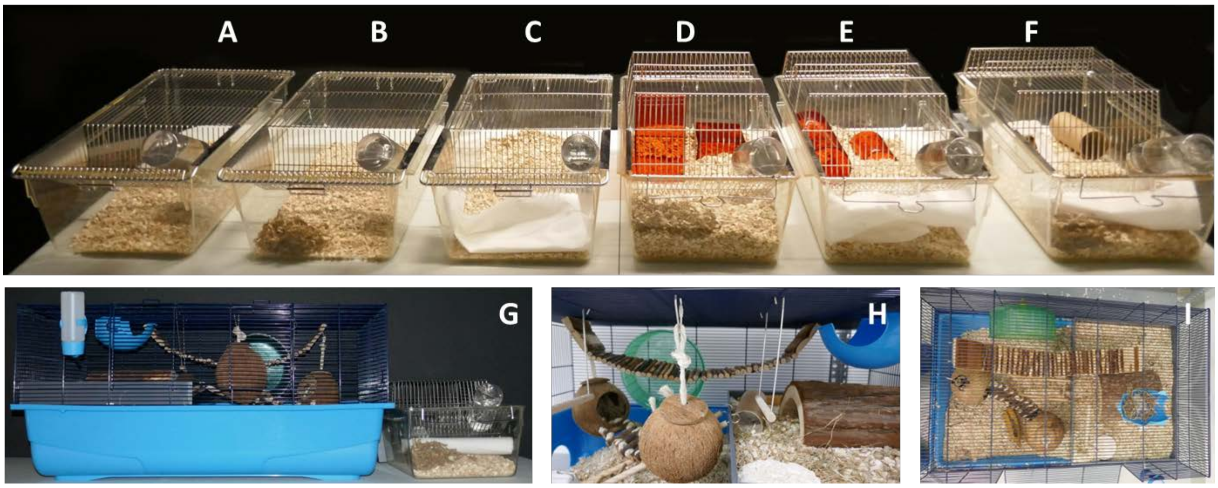

Supplementary Figure 3: Pictures of rotated items in the Super–Enriched. A) kapok; B) rope ladder; C) salt lick with herbs; D) running wheel; E) wooden bridge; F) trapeze; G) aspen bricks.

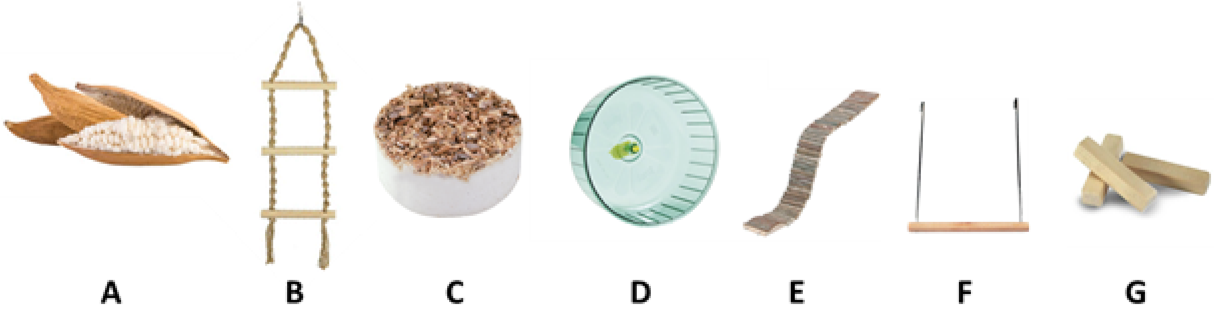

Supplementary Figure 4. Guessing task apparatus. A) back wall with goal holes; B) apparatus with guillotine doors closed; C) apparatus with guillotine doors open

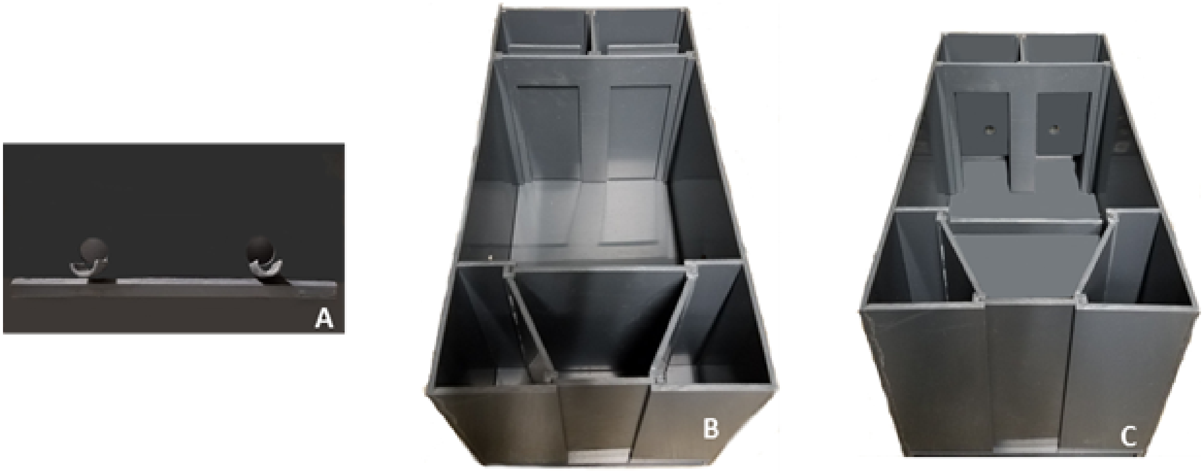

Supplementary Figure 5. Testing timeline for a single cohort in the guessing task

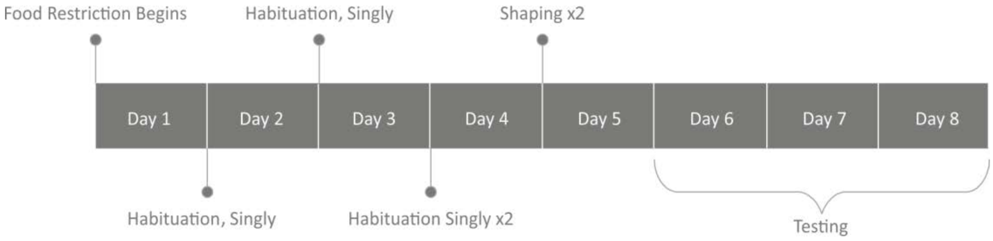

Supplementary Figure 6. Judgement bias apparatus. A) the row of holes or goal-boxes that served as the spatial cue when training and testing mice. Only five of the holes were used in the current study: every second hole starting from the outmost hole. B) Overview of the apparatus with the goal-holes at the top and the nose poke apparatus used as trial initiator at the bottom.

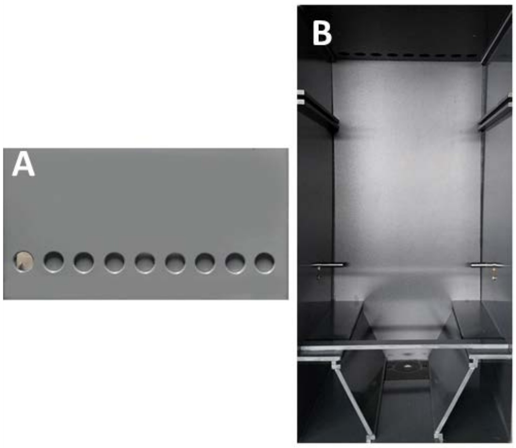

Supplementary Figure 7. Proportion of active intervals (Median and IQR) where mice were engaged in different forms of stereotypic behavior in relation to housing condition. Note, the y-axis is truncated to 40% from 100% to aid with visual clarity, given the low levels of stereotypic behavior. Shaded circles and stars represent outliers, and are designated per SPSS convention; shaded circles are those values between the inner and outer fence while stars represent values beyond the outer fence.

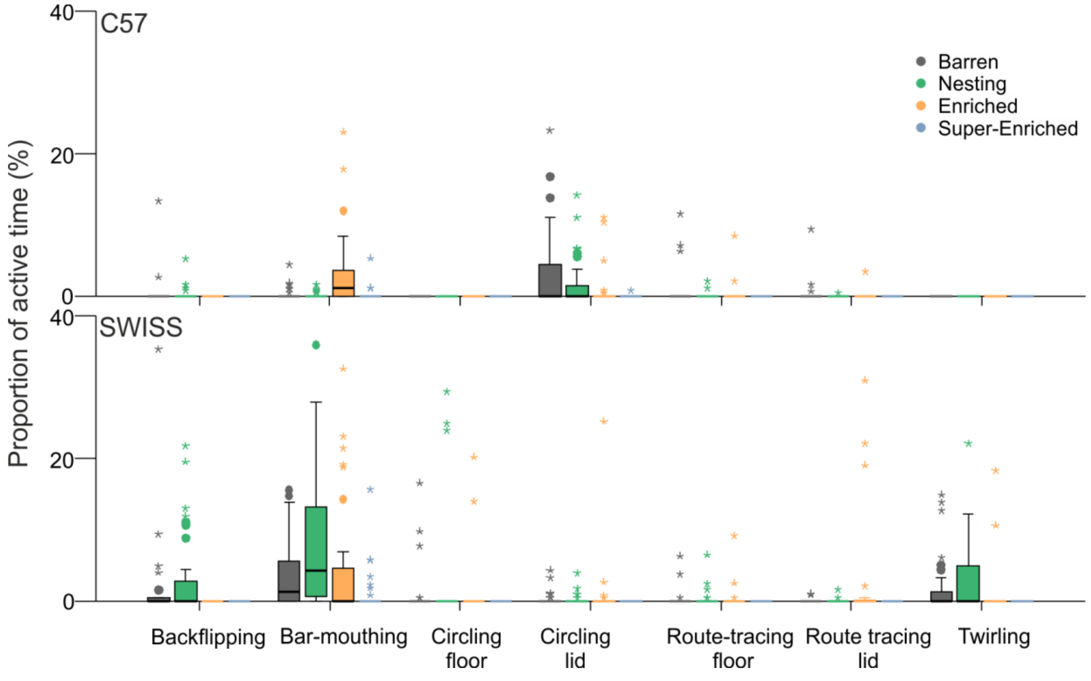

Supplement: Supplementary file 1 [file Presentation_1.pdf]
